# Role of Therapeutic Anticoagulation in COVID-19: The Current Situation

**DOI:** 10.3390/hematolrep15020037

**Published:** 2023-06-05

**Authors:** Mandeep Singh Rahi, Jay Parekh, Prachi Pednekar, Mayuri Mudgal, Vishal Jindal, Kulothungan Gunasekaran

**Affiliations:** 1 Division of Pulmonary Diseases and Critical Care Medicine, Yale-New Haven Health Lawrence and Memorial Hospital, New London, CT 06320, USA; sunny.mandeep@gmail.com; 2 Department of Internal Medicine, Yale-New Haven Health Bridgeport Hospital, Bridgeport, CT 06610, USA; 3 Department of Internal Medicine, Yale-New Haven Hospital, New Haven, CT 06510, USA; 4 Department of Medicine, Camden Clark Medical Center, Parkersburg, WV 26101, USA; 5 Department of Hematology and Oncology, Sinai Hospital, Baltimore, MD 21215, USA; 6 Department of Pulmonary and Critical Care, Yuma Regional Medical Center, Yuma, AZ 85364, USA

**Keywords:** COVID-19, thrombosis, coagulopathy, anticoagulation, heparin, mortality

## Abstract

Thrombotic complications from COVID-19 are now well known and contribute to significant morbidity and mortality. Different variants confer varying risks of thrombotic complications. Heparin has anti-inflammatory and antiviral effects. Due to its non-anticoagulant effects, escalated-dose anticoagulation, especially therapeutic-dose heparin, has been studied for thromboprophylaxis in hospitalized patients with COVID-19. Few randomized, controlled trials have examined the role of therapeutic anticoagulation in moderately to severely ill patients with COVID-19. Most of these patients had elevated D-dimers and low bleeding risks. Some trials used an innovative adaptive multiplatform with Bayesian analysis to answer this critical question promptly. All the trials were open-label and had several limitations. Most trials showed improvements in the meaningful clinical outcomes of organ-support-free days and reductions in thrombotic events, mainly in non-critically-ill COVID-19 patients. However, the mortality benefit needed to be more consistent. A recent meta-analysis confirmed the results. Multiple centers initially adopted intermediate-dose thromboprophylaxis, but the studies failed to show meaningful benefits. Given the new evidence, significant societies have suggested therapeutic anticoagulation in carefully selected patients who are moderately ill and do not require an intensive-care-unit level of care. There are multiple ongoing trials globally to further our understanding of therapeutic-dose thromboprophylaxis in hospitalized patients with COVID-19. In this review, we aim to summarize the current evidence regarding the use of anticoagulation in patients with COVID-19 infection.

## 1. Introduction

**Figure 1:**
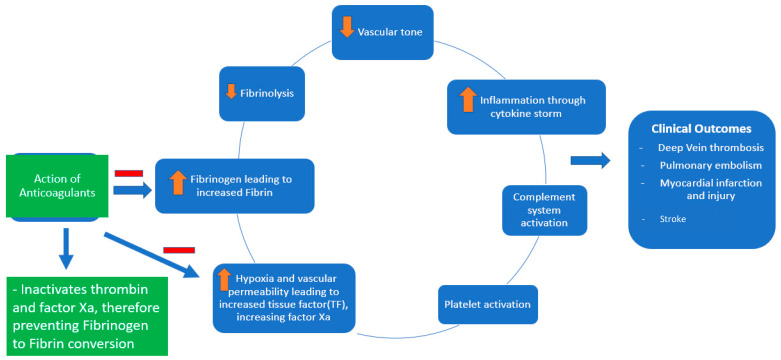
Pathophysiology of COVID-19-associated coagulopathy and the role of anticoagulants (heparin and low-molecular-weight heparin).

Coagulopathy and thrombosis are now well known complications of COVID-19, contributing to significant morbidity and mortality [1,2]. The pathogenesis of the coagulopathy associated with COVID-19 is complex (Figure 1). It involves macrophage activation, cytokine storm, platelet activation, and endothelial cell activation, eventually activating the intrinsic and extrinsic coagulation pathways [2]. COVID-19 causes DIC, which differs from the typical septic DIC, with less bleeding and elevated fibrinogen levels [1]. Thromboprophylaxis is indispensable in hospitalized patients with COVID-19. There has been significant interest in defining the role of therapeutic-dose anticoagulation, especially with heparin, in patients acutely ill with COVID-19. Although well known for its anticoagulant activity, heparin, either unfractionated heparin (UFH) or low-molecular-weight heparin (LMWH), has various other pleiotropic effects [3].

Heparin has been shown to exhibit anti-inflammatory and antiviral properties (at a dose of 500–1000 μg/mL heparin in Vero E6 cells) [4,5]. Soluble heparin interacts with the SARS-CoV-2 spike protein and impairs its entry into the host cells [6]. Heparin exerts its anti-inflammatory properties by binding to and inhibiting chemokines, cytokines, and complement, growth, and angiogenic factors. It also prevents endothelial dysfunction and vascular injury by binding to adhesion molecules during inflammation. Heparin reduces vascular leak injury by decreasing thrombin formation [5].

The above-mentioned anti-inflammatory effects (notably, the decreased levels of IL-6, IL-8, and inflammatory biomarkers of COVID-19-CRP and procalcitonin) are also noted with prophylactic doses of LMWH at 40 mg daily [7]. Heparin has been investigated as a therapeutic agent in various inflammatory conditions such as chronic obstructive pulmonary disease (COPD), cystic fibrosis, and sepsis [3,4]. This review provides an overview of the most current evidence and recommendations on using therapeutic-dose anticoagulation in patients acutely ill with COVID-19. For this review, therapeutic-dose anticoagulation means therapeutic-dose heparin (UFH or LMWH), unless otherwise specified.

## 2. How Does the Omicron Variant Compare to the Other COVID-19 Variants with Hyper-Coagulopathy?

In November 2021, the South African Ministry of Health reported the emergence of a new variant of SARS-CoV-2, called omicron. Soon, the WHO designated this novel variant a SARS-CoV-2 variant of concern (the others being alpha, beta, gamma, and delta), which denotes a variant with evidence of an increase in transmissibility, more severe disease (increased hospitalizations or deaths), a significant reduction in neutralization by antibodies generated during a previous infection or vaccination, reduced effectiveness of treatments or vaccines, or diagnostic detection failures [8]. Currently, omicron (the B.1.1.529, BA.1, BA.1.1, BA.2, BA.3, BA.4, and BA.5 lineages) remains the only variant of concern in the USA as classified by the US government SARS-CoV-2 Interagency Group (SIG) [9].

Omicron and its subvariants have been known to have more than thirty mutations in the spike protein, causing higher infectivity (by strengthening the binding with host ACE2 receptors) and reduced neutralization by antibodies. Omicron primarily infects the upper respiratory tract compared with the other variants, which affect the lower respiratory tract and cause a milder disease [10]. A study from South Africa published as a research letter noted that most of the omicron-infected COVID-19 patients were unvaccinated (66.4%) and were less likely to require oxygen therapy or mechanical ventilation, or to need ICU admission, therefore denoting relatively low disease severity [11]. They grouped the data as wave 1 (14 June–6 July 2020), wave 2 (1–23 December 2020), and wave 3 (1–23 June 2021), and compared them with the fourth wave (15 November–7 December 2021). The median length of stay and mortality rates were also low in the fourth wave despite a rapid surge of COVID-19 cases [11]. The impact of SARS-CoV-2 genome mutations on COVID-19-associated coagulopathy has not been well documented.

Interestingly, a study compared whole-blood thrombo-elastography (TEG^®^) blood clotting parameters and the prevalence of micro-clots in healthy individuals and in COVID-19 patients that different SARS-CoV-2 variants had infected. This study noted that most of the patients infected with the beta and delta variants were sicker compared to those infected with the omicron variants. The study indicated lesser hypercoagulability with the omicron variant compared with healthy individuals versus the beta/delta variant population when compared with the same healthy individuals. A direct comparison between the omicron and the beta/delta variant populations indicated a significantly higher maximum amplitude with the latter. Similarly, the plasma micro-clot analysis noted a significant amount of fibrin amyloid micro-clots with the beta/delta variants compared with the omicron variant [12].

## 3. Therapeutic-Dose Thromboprophylaxis

**Figure 2:**
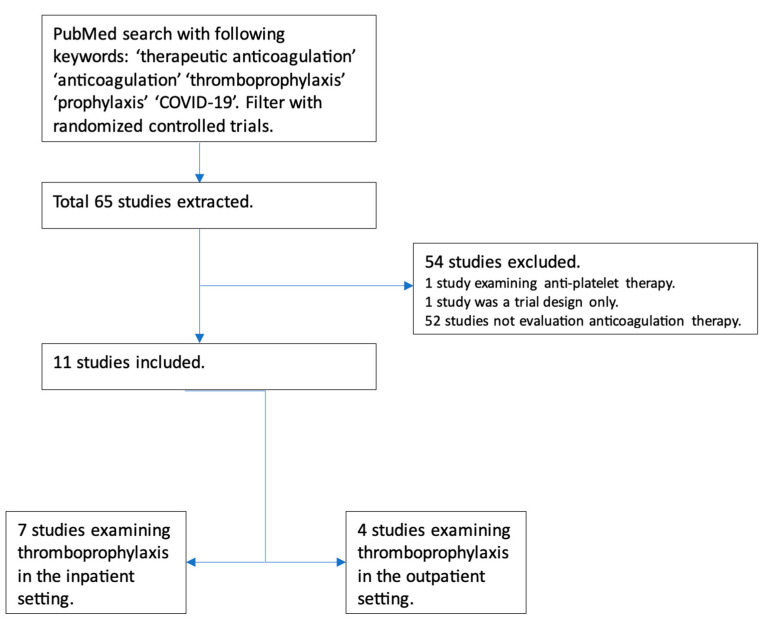
Flowchart depicting trials examining the role of full-dose anticoagulation as prophylaxis in patients with COVID-19 infection.

Initial data on the use of anticoagulation in COVID-19 came from China in March 2020, involving 449 patients, out of which 94 received anticoagulation. Still, none of these patients received full-dose anticoagulation [13]. In July 2020, a retrospective analysis was conducted among patients with COVID-19 admitted to a particular health system in New York. This analysis included about 2700 patients with COVID-19. While the exact reason for anticoagulation was unclear among these patients, on multivariate analysis, the study demonstrated survival benefits among the patients who received full-dose anticoagulation compared with those who did not. While it did adjust for prior anticoagulation use before hospitalization for other causes, the study had several limitations. Still, it raised an essential question regarding using full-dose anticoagulation [14]. We identified the clinical trials examining the role of full-dose anticoagulation in patients with COVID-19 infection (Figure 2).

Seven major randomized controlled trials have examined the role of therapeutic-dose anticoagulation in patients with COVID-19. These included patients with at least moderate COVID-19 infection, elevated D-dimer levels, and low bleeding risk. Significant limitations of these trials were the lack of a standardized definition of disease severity and varied anticoagulation regimens in terms of agents (DOACs and heparin); the duration of anticoagulation, time to randomization, and duration of follow-up varied significantly as well. Furthermore, all had an open-label design, introducing the risk of bias. Standard-of-care thromboprophylaxis practices in the control patients differed; some received intermediate-dose thromboprophylaxis. Despite the limitations, these trials were conducted during the difficult time of an ongoing pandemic and provided invaluable insight into the role of therapeutic-dose anticoagulation, mainly heparin, in hospitalized COVID-19 patients.

In September 2020, a phase 2 trial compared empiric anticoagulation with standard-dose thromboprophylaxis among patients with COVID-19 and ARDS who required mechanical ventilation. Although only ten patients were included in each arm, they found a statistically significant improvement in the blood gas exchange in addition to a decreased need for mechanical ventilation among the therapeutic anticoagulation group [15]. Another propensity-matched analysis of COVID-19 patients showed a mortality benefit among intubated patients but not in non-critically-ill hospitalized patients [16]. The earliest randomized controlled data came from an open-label trial in Brazil that used the therapeutic-dose rivaroxaban or enoxaparin for anticoagulation in COVID-19 admitted patients. The trial included hospitalized patients with elevated D-dimers, with at least a third of the patients in both groups having severe disease. The study did not reveal a statistical difference in the primary composite outcome, including mortality, duration of hospitalization, or period of oxygen needed to day 30. Still, regarding safety outcomes, there was a higher incidence of bleeding events in the therapeutic anticoagulation group [17]. This trial included patients who had symptoms up to 14 days before randomization with a median time of randomization of day 10. Although >80% of patients had moderate disease at baseline, only a quarter had markedly elevated D-dimer (>3 × ULN). A significant difference in this trial was DOAC, which may not have the same non-anticoagulant effects as heparin does.

In August 2021, *NEJM* published a large multicenter international randomized controlled trial investigating therapeutic-dose anticoagulation in COVID. The investigators stratified patients into moderate vs. severe disease based on ICU requirements and published the results separately. In the moderate disease subgroup, they found therapeutic anticoagulation, compared with prophylactic anticoagulation, was associated with more organ-support-free days, which was statistically significant regardless of the patient’s baseline D-dimer level. There was no significant difference in the risk of major bleeding [18]. The same study reported outcomes of the severe subgroup separately, revealing that therapeutic anticoagulation did not result in a statistically significant difference in organ-support-free survival days [19]. This difference in results was intriguing and there is a possibility that the benefit of anticoagulation may be present only in the initial period of the disease, which would potentially explain the difference in results. There might also be inherent differences among the population developing severe disease, making therapeutic heparin less beneficial. These trials used the new innovative response-adaptative randomization and complex Bayesian analysis. They included clinically meaningful outcomes of organ-support-free days, mortality, and the need for intubation in addition to the incidence of VTE. Some of the limitations of these trials include the risk of confirmation bias given the open-label design; more than 70% of patients were excluded due to the stringent exclusion criteria, which varied among the three trial platforms, diminishing the generalizability of the results.

Around 28% of patients in the control group received higher than standard doses of thromboprophylaxis, and 20% of patients in the experimental group did not receive therapeutic doses of heparin. Only 36% of the patients received remdesivir, 60% received glucocorticoids, and less than 1% received tocilizumab, deviating from the current standard of care with high usage of glucocorticoids and remdesivir early in the disease course [19,20]. The RAPID trial was another adaptive multicenter open-label randomized controlled trial of 465 patients with COVID-19 and elevated D-dimer who were hospitalized in a non-ICU level of care setting. Although the primary composite outcomes of death, invasive or noninvasive mechanical ventilation, or ICU admission did not differ, the all-cause mortality was significantly lower in the therapeutic anticoagulation group (1.8% vs. 7.6%, OR 0.22, 95% CI 0.07 to 0.65; *p* = 0.006). No increase in major bleeding was noted in the therapeutic anticoagulation group [21].

A recent meta-analysis that included over 5000 patients found that escalated-dose prophylactic anticoagulation (intermediate or therapeutic dose) did not reduce all-cause mortality when compared with standard-dose prophylactic anticoagulation (17.8% vs. 18.6%; risk ratio (RR) 0.96, 95% CI 0.78–1.18). Escalated-dose prophylactic anticoagulation decreased the rates of VTE (2.5% vs. 4.7%; RR 0.55, 95% CI 0.41–0.74) with a number needed to treat (NNT) of 46 but increased the risk of major bleeding (2.4% vs. 1.4%; RR 1.73, 95% CI 1.15–2.60) with a number needed to harm (NNH) of 102. Results did not differ for the subgroups of critically ill and non-critically ill patients. An interesting finding from the meta-analysis was that the median time to randomization was ten days, which one can argue might be late to have benefited from the non-anticoagulation properties of heparin [22]. In contrast, another systematic review included 23 retrospective studies and over 25,000 patients. Therapeutic anticoagulation reduced mortality (RR 0.30, 95% CI 0.15–0.60; I^2^ 58%) but increased the risk of bleeding (RR 2.53, 95% CI 1.60–4.00; I^2^ 58%). The results should be interpreted with caution. All the included studies were observational, and the subgroup analysis had a high degree of heterogeneity [23]. Table 1 outlines the major randomized controlled trials examining the effects of therapeutic-dose anticoagulation.

The INSPIRATION trial examined the role of intermediate-dose thromboprophylaxis compared with a standard dose in 562 critically ill patients with COVID-19. There was no difference in the composite outcome of venous or arterial thrombosis, treatment with ECMO, or 30-day mortality. The intermediate-dosing group had statistically significant thrombocytopenia but without increased risk of major bleeding [24]. Following this, Perepu et al. analyzed 176 patients with COVID-19 who were critically ill and received either intermediate-dose or standard-dose thromboprophylaxis. There were no differences in all-cause mortality, thrombotic complications, or major bleeding events [25]. In a recent trial, rivaroxaban was superior to prophylactic enoxaparin in preventing thrombotic events with less bleeding in patients with mild to moderate COVID-19 infection. This study included only 230 patients, limiting the generalizability [26].

The role of anticoagulation has also been studied in outpatients, given the concern for thrombosis in this subgroup of patients. The ACTIV 4B trial compared antithrombotics and anticoagulants in symptomatic COVID-19 patients in the outpatient setting. The study included four groups: low-dose aspirin, 2.5 mg apixaban, 5 mg apixaban, and placebo. All groups had similar primary outcomes: a composite of all-cause mortality, symptomatic venous or arterial thromboembolism, and hospitalization from pulmonary and cardiovascular events, including myocardial infarction and stroke. There were no major bleeding events [27]. In another multicenter trial from Brazil, rivaroxaban at discharge in patients at high risk for venous thromboembolism reduced the risk of venous or arterial thromboembolic events and cardiovascular death on day 35 [28]. The ETHIC trial, examining the role of LMWH in unvaccinated outpatients with COVID-19, was stopped early due to slow enrollment and low event rates. It suggested no benefit of using LMWH [29]. Another prospective trial examining the role of post-discharge thromboprophylaxis with apixaban 2.5 mg twice daily was inconclusive, as the study was stopped early due to a low event rate [30].

**Table 1:** Major randomized controlled trials examining the effects of therapeutic-dose anticoagulation vs. standard-dose anticoagulation in patients with COVID-19.

The Study, Author (Year)	Study Type	Patients	Study Drug 1	Comparator	Efficacy Outcomes	Safety Outcomes
HESACOVID trial, Lemos et al. (2020) [15]	Phase II randomized, controlled trial	20 patients requiring mechanical ventilation	Therapeutic-dose enoxaparin	Standard anticoagulant prophylaxis	Statistically significant increase in P/F ratio at 7 days and 14 days. A higher proportion of successful extubation and more ventilator-free days in the therapeutic anticoagulation group.	No major or minor bleeding events. No difference in bleeding events requiring medical attention between the groups.
The REMAP-CAP, ACTIV-4a, ATTACC trial, Goligher et al. (2021) [19]	Open-label, adaptive, multiplatform randomized, controlled trial	1098 critically ill patients with severe COVID-19 pneumonia	Therapeutic-dose anticoagulation	Standard of care thromboprophylaxis	Median organ-support-free days were 1 vs. 4 in the standard care group. A similar proportion of patients in both groups survived hospital discharge (62.7% vs. 64.5%).	Major bleeding rates were 3.8% in the intervention group and 2.3% in the standard-of-care group.
The ATTACC, ACTIV-4a, REMAP-CAP trial, Lawler et al. (2021) [18]	Open-label, adaptive, multiplatform randomized, controlled trial	2219 non-critically-ill (moderately ill, not requiring ICU level of care) patients	Therapeutic-dose anticoagulation (*n* = 1190)	Standard of care thromboprophylaxis (*n* = 1054)	The probability of increasing OSFD with therapeutic-dose anticoagulation was 98.6% compared with standard-of-care thromboprophylaxis (Adjusted OR 1.27, 95% CI 1.03 to 1.58). The therapeutic AC arm had a higher probability of survival until hospital discharge.	Major bleeding was not statistically significant in the two groups (1.9% vs. 0.9%).
RAPID trial, Sholzberg et al. (2021) [21]	Open-label, adaptive, randomized, controlled trial	465 moderately ill patients (not in ICU) and increased D-dimer levels	Therapeutic-dose heparin (*n* = 228)	Prophylactic dose (standard or intermediate dosing) heparin (*n* = 237)	The primary outcome was a composite of death, invasive and noninvasive mechanical ventilation, or ICU admission up to day 28, which occurred in 16.2% of patients in the experimental arm and 21.9% in the control arm (OR 0.69, 95% CI 0.43 to 1.10; *p* = 0.12). Death occurred in 1.8% vs. 7.6% in the control group (OR 0.22, 95% CI 0.07 to 0.65; *p* = 0.006). VTE rates were not different.	Major bleeding rates were not statistically significant in the two groups (0.9% vs. 1.7%).
HEP-COVID trial, Spyropoulos et al. (2021) [31]	Multicenter, randomized, controlled trial	253 patients hospitalized with COVID-19 and elevated D-dimer levels (>4 × ULN)	Therapeutic-dose enoxaparin (*n* = 129)	Institutional standard- or intermediate-dose thromboprophylaxis (*n* = 124)	The primary outcome was a composite of VTE, ATE, or death from any cause, which occurred in 29% in the therapeutic group vs. 42% in the standard of care group (RR 0.68, 95% CI 0.49 to 0.96; *p* = 0.03). This benefit was not seen in the ICU group of patients.	Major bleeding occurred in 5% vs. 2% in the standard of care group (RR 2.88; 95% CI 0.59 to 14.02; *p* = 0.17).
ACTION trial, Lopes et al. (2021) [17]	Open-label, pragmatic, randomized, controlled trial	615 patients with COVID-19 and elevated D-dimer	Therapeutic-dose rivaroxaban in stable patients or heparin (UFH or LMWH) followed by rivaroxaban to day 30 (*n* = 311)	Institutional prophylactic anticoagulation with heparin (UFH or LMWH) (*n* = 304)	The primary outcome of the time of death, duration of hospitalization, or duration of supplemental oxygen to day 30 was not different in the two groups.	Higher major or CRNMB was seen in the therapeutic group at 8% vs. 2% in the standard group (RR 3.64, 95% CI 1.61 to 8.27; *p* = 0.0010).

Abbreviations: COVID-19, Coronavirus Disease 2019; UFH, unfractionated heparin; LMWH, low-molecular-weight heparin; CRNMB, clinically relevant non-major bleeding events; ICU, intensive care unit; ULN, the upper limit of normal; VTE, venous thromboembolism; ATE, arterial thromboembolism; OSFD, organ-support-free days; P/F, the ratio of partial pressure of oxygen and fraction of inspired oxygen.

## 4. Management Recommendations

Given the evidence of macro- and micro-thrombosis in patients with COVID-19, emphasis was placed on intermediate-dose thromboprophylaxis. Although not backed by solid evidence, multiple institutions developed their policies on thromboprophylaxis using D-dimer levels and weight-based dosing. One approach used a three-tier stratification of COVID-19 patients according to the D-dimer, creatinine clearance, and BMI. These were divided into standard-, intermediate-, and therapeutic-dose groups [32]. More recent evidence suggests intermediate dosing might not be helpful [22,24].

Given the current evidence, most major society guidelines suggest using therapeutic-dose anticoagulation, mainly with heparin (UFH or LMWH), in non-critically-ill patients with COVID-19 who have elevated D-dimer levels and are at low bleeding risk. Table 2 lists the recommendations from major society guidelines on using therapeutic-dose anticoagulation in patients with COVID-19. It is important to note that most of these recommendations are weak or conditional and based on low-quality evidence. The use of therapeutic-dose anticoagulation should be evaluated carefully in each patient with COVID-19. Their demographics (age, sex, race), underlying co-morbidities, SARS-CoV-2 variant form, vaccination status, cardiorespiratory reserve status, and bleeding risk should be considered. Clinicians can use various scoring tools to calculate the bleeding risk [33]. A high index of suspicion should be maintained for any thrombotic (VTE, stroke) and bleeding complications (gastrointestinal bleeding or closed-space bleeding such as retroperitoneal, intracerebral, and intercostal artery bleeding) [34].

## 5. Conclusions

COVID-19 remains a global public health issue, although the infection rate is declining due to widespread vaccination. Despite breakthroughs in vaccinations and therapeutics, a significant population worldwide remains at risk for morbidity and mortality. Thrombotic complications are well known, and the role of thromboprophylaxis cannot be overstated. Therapeutic-dose anticoagulation should be considered carefully in hospitalized patients with COVID-19. There has been significant progress in recent trials, but clinicians should consider their limitations curtailing generalizability and the varied manifestations of this disease [20]. Therapeutic anticoagulation has not convincingly shown mortality benefits but significantly reduces the rate of VTE during COVID. Even though the trials used stringent inclusion criteria, some had higher rates of major bleeding [22,23]. The right time for initiating anticoagulation during the disease course remains to be elucidated, but the benefit appears early in the disease. Multiple ongoing trials worldwide evaluate the role of escalated-dose anticoagulation in patients with COVID-19 (Table 3). Hopefully, these studies will clarify some of the unanswered questions.

## Figures and Tables

**Table 2:** Current major society recommendations on therapeutic-dose anticoagulation for thromboprophylaxis in patients with COVID-19.

Society (Year of Publication)	Recommendations	Level of Recommendation and Evidence
American Society of Hematology (2022) [35]	Recommends using therapeutic-intensity over prophylactic-intensity anticoagulation in patients with non-critically-ill COVID-19 infection who do not have suspected or confirmed VTE or another indication for anticoagulation.	Conditional recommendation, very low certainty in evidence
International Society on Thrombosis and Haemostasis (2022) [36]	In select non-critically-ill patients hospitalized for COVID-19, therapeutic-dose LMWH or UFH is beneficial in preference to low- (prophylactic) or intermediate-dose LMWH or UFH to reduce the risk for thromboembolism and end-organ failure.	Strong, high-quality evidence
	In critically ill patients hospitalized with COVID-19, therapeutic-dose LMWH/UFH is not recommended over the usual care or prophylactic-dose LMWH/UFHs.	No benefit, moderate-quality evidence
The Anticoagulation Forum (2022) [37]	Suggests considering therapeutic intensity anticoagulation (LMWH or UFH) in non-critically-ill patients at increased risk of disease progression (hypoxic, elevated D-dimer) and not at increased risk of bleeding.	Less strong evidence
	Recommends using standard-dose thromboprophylaxis instead of intermediate or therapeutic intensity thromboprophylaxis in critically ill patients.	Strong evidence
National Institutes of Health (2022) [38]	Recommends therapeutic-dose heparin for hospitalized patients with COVID-19 pneumonia who require supplemental oxygen through low-flow nasal cannula and are non-pregnant, with elevated D-dimer, and not at increased bleeding risk.	Weak recommendation and level IIa quality of evidence
	Recommends prophylactic-dose heparin in critically ill patients with COVID-19 pneumonia (those requiring supplemental oxygen through a high-flow device or NIV, requiring MV).	Strong recommendation and level I quality of evidence
American College of Chest Physicians (2022) [39]	Suggests therapeutic-dose heparin (LMWH or UFH) over standard-dose anticoagulant thromboprophylaxis in acutely ill patients with COVID-19 pneumonia and low risk of bleeding.	Conditional recommendation, Ungraded Consensus-Based Statement
	Suggests standard-dose anticoagulant thromboprophylaxis over therapeutic- or intermediate-dose anticoagulation in critically ill patients with COVID-19 pneumonia.	Conditional recommendation, Ungraded Consensus-Based Statement

Abbreviations: COVID-19, Coronavirus Disease 2019; UFH, unfractionated heparin; LMWH, low-molecular-weight heparin; VTE, venous thromboembolism; NIV, noninvasive ventilation; MV, mechanical ventilation.

**Table 3:** Ongoing trials evaluating the role of escalated-dose (therapeutic or intermediate) anticoagulation in patients with COVID-19.

Study	Intervention	Number of Patients	Outcomes
NCT04445935	Comparing therapeutic-dose anticoagulation with bivalirudin with standard thromboprophylaxis	100 patients with severe COVID-19 pneumonia requiring mechanical ventilation and with elevated D-dimer (1.2 mg/L)	Change in P/F ratio at 3 days and change in kidney function at 3 days
NCT04808882	Comparing low-dose, high-dose prophylactic tinzaparin and therapeutic tinzaparin	353 patients with severe COVID-19 pneumonia	All-cause mortality and number of days to clinical improvement
NCT04377997	Comparing therapeutic-dose anticoagulation with the standard of care	300 patients with COVID-19 pneumonia and elevated D-dimer (>1.5 g/mL)	Composite efficacy end point of death, cardiac arrest, VTE, MI, and shock in 12 weeks
NCT04646655	Comparing therapeutic with prophylactic dosing enoxaparin	300 patients with COVID-19 pneumonia, moderate-severe ARDS, and elevated D-dimer (>2000 ng/mL)	Mortality and progression of respiratory failure at 30 days; major bleeding episodes
NCT04584580	Comparing therapeutic-dose LMWH with D-dimer-adjusted LMWH dosing	500 patients with severe COVID-19 pneumonia	Mortality and occurrence of arterial and/or venous thrombosis at 4 weeks or discharge
NCT04406389	Comparing intermediate-dose prophylaxis with therapeutic-dose anticoagulation	186 patients with moderate to severe COVID-19 pneumonia and elevated D-dimer (>700 mg/mL)	Mortality at 30 days, ICU length of stay, number of arterial and/or venous thrombotic events, number of bleeding events
NCT04512079	Comparing prophylactic-dose and full-dose enoxaparin with full-dose apixaban	3600 patients admitted with COVID-19 pneumonia and elevated D-dimer	The time to first event rate within 30 days of randomization of the composite of all-cause mortality, intubation requiring mechanical ventilation, and VTE
NCT04505774	Comparing therapeutic- and prophylactic-dose anticoagulation alone versus the addition of a P2Y12 inhibitor	3000 patients with moderate to severe COVID-19 pneumonia	Organ-support-free days and all-cause mortality
NCT04508023	Comparing full-dose rivaroxaban with the standard of care	4000 patients with acute symptomatic COVID-19 infection not requiring hospitalization	Time to first occurrence of a composite endpoint of symptomatic VTE, MI, ischemic stroke, acute limb ischemia, non-CNS systemic embolization, all-cause hospitalization, and all-cause mortality

Abbreviations: COVID-19, Coronavirus Disease 2019; LMWH, low-molecular-weight heparin; VTE, venous thromboembolism; MI, myocardial infarction; CNS, central nervous system; ICU, intensive care unit.

## Data Availability

Not applicable.

## References

[1] Asakura H., Ogawa H. (2021). COVID-19-associated coagulopathy and disseminated intravascular coagulation. Int. J. Hematol..

[2] Rahi M.S., Jindal V., Reyes S.-P., Gunasekaran K., Gupta R., Jaiyesimi I. (2021). Hematologic disorders associated with COVID-19: A review. Ann. Hematol..

[3] Tritschler T., Le Gal G., Brosnahan S., Carrier M. (2022). POINT: Should Therapeutic Heparin Be Administered to Acutely Ill Hospitalized Patients with COVID-19? Yes. Chest.

[4] Conzelmann C., Müller J.A., Perkhofer L., Sparrer K.M., Zelikin A.N., Münch J., Kleger A. (2020). Inhaled and systemic heparin as a repurposed direct antiviral drug for prevention and treatment of COVID-19. Clin. Med. Lond.

[5] Cassinelli G., Naggi A. (2016). Old and new applications of non-anticoagulant heparin. Int. J. Cardiol..

[6] Kim S.Y., Jin W., Sood A., Montgomery D.W., Grant O.C., Fuster M.M., Fu L., Dordick J.S., Woods R.J., Zhang F. (2020). Characterization of heparin and severe acute respiratory syndrome-related coronavirus 2 (SARS-CoV-2) spike glycoprotein binding interactions. Antivir. Res..

[7] Saithong S., Saisorn W., Tovichayathamrong P., Filbertine G., Torvorapanit P., Wright H.L., Edwards S.W., Leelahavanichkul A., Hirankarn N., Chiewchengchol D. (2022). Anti-Inflammatory Effects and Decreased Formation of Neutrophil Extracellular Traps by Enoxaparin in COVID-19 Patients. Int. J. Mol. Sci..

[8] Classification of Omicron (B.1.1.529): SARS-CoV-2 Variant of Concern. https://www.who.int/news/item/26-11-2021-classification-of-omicron-(b.1.1.529)-sars-cov-2-variant-of-concern.

[9] SARS-CoV-2 Variant Classifications and Definitions. https://www.cdc.gov/coronavirus/2019-ncov/variants/variant-classifications.html#anchor_1632154493691.

[10] Halfmann P.J., Iida S., Iwatsuki-Horimoto K., Maemura T., Kiso M., Scheaffer S.M., Darling T.L., Joshi A., Loeber S., Singh G. (2022). SARS-CoV-2 Omicron virus causes attenuated disease in mice and hamsters. Nature.

[11] Maslo C., Friedland R., Toubkin M., Laubscher A., Akaloo T., Kama B. (2022). Characteristics and Outcomes of Hospitalized Patients in South Africa during the COVID-19 Omicron Wave Compared with Previous Waves. JAMA.

[12] Grobbelaar L.M., Kruger A., Venter C., Burger E.M., Laubscher G.J., Maponga T.G., Kotze M.J., Kwaan H.C., Miller J.B., Fulkerson D. (2022). Relative Hypercoagulopathy of the SARS-CoV-2 Beta and Delta Variants when Compared to the Less Severe Omicron Variants Is Related to TEG Parameters, the Extent of Fibrin Amyloid Microclots, and the Severity of Clinical Illness. Semin. Thromb. Hemost..

[13] Tang N., Bai H., Chen X., Gong J., Li D., Sun Z. (2020). Anticoagulant treatment is associated with decreased mortality in severe coronavirus disease 2019 patients with coagulopathy. J. Thromb. Haemost..

[14] Paranjpe I., Fuster V., Lala A., Russak A.J., Glicksberg B.S., Levin M.A., Charney A.W., Narula J., Fayad Z.A., Bagiella E. (2020). Association of Treatment Dose Anticoagulation with In-Hospital Survival Among Hospitalized Patients With COVID-19. J. Am. Coll. Cardiol..

[15] Lemos A.C.B., do Espírito Santo D.A., Salvetti M.C., Gilio R.N., Agra L.B., Pazin-Filho A., Miranda C.H. (2020). Therapeutic versus prophylactic anticoagulation for severe COVID-19: A randomized phase II clinical trial (HESACOVID). Thromb. Res..

[16] Yu B., Gutierrez V.P., Carlos A., Hoge G., Pillai A., Kelly J.D., Menon V. (2021). Empiric use of anticoagulation in hospitalized patients with COVID-19: A propensity score-matched study of risks and benefits. Biomark. Res..

[17] Lopes R.D., de Barros E Silva P.G.M., Furtado R.H.M., Macedo A.V.S., Bronhara B., Damiani L.P., Barbosa L.M., de Aveiro Morata J., Ramacciotti E., de Aquino Martins P. (2021). Therapeutic versus prophylactic anticoagulation for patients admitted to hospital with COVID-19 and elevated D-dimer concentration (ACTION): An open-label, multicentre, randomised, controlled trial. Lancet.

[18] Lawler P.R., Goligher E.C., Berger J.S., Neal M.D., McVerry B.J., Nicolau J.C., Gong M.N., Carrier M., Rosenson R.S., Reynolds H.R. (2021). Therapeutic Anticoagulation with Heparin in Noncritically Ill Patients with COVID-19. N. Engl. J. Med..

[19] Goligher E.C., Bradbury C.A., McVerry B.J., Lawler P.R., Berger J.S., Gong M.N., Carrier M., Reynolds H.R., Kumar A., Turgeon A.F. (2021). Therapeutic Anticoagulation with Heparin in Critically Ill Patients with COVID-19. N. Engl. J. Med..

[20] Jimenez D., Rali P., Doerschug K. (2022). COUNTERPOINT: Should Therapeutic Heparin Be Administered to Acutely Ill Hospitalized Patients with COVID-19? No. Chest.

[21] Sholzberg M., Tang G.H., Rahhal H., AlHamzah M., Kreuziger L.B., Áinle F.N., Alomran F., Alayed K., Alsheef M., AlSumait F. (2021). Effectiveness of therapeutic heparin versus prophylactic heparin on death, mechanical ventilation, or intensive care unit admission in moderately ill patients with COVID-19 admitted to hospital: RAPID randomised clinical trial. Bmj.

[22] Ortega-Paz L., Galli M., Capodanno D., Franchi F., Rollini F., Bikdeli B., Mehran R., Montalescot G., Gibson C.M., Lopes R.D. (2021). Safety and efficacy of different prophylactic anticoagulation dosing regimens in critically and non-critically ill patients with COVID-19: A systematic review and meta-analysis of randomized controlled trials. Eur. Heart J. Cardiovasc. Pharmacother..

[23] Parisi R., Costanzo S., Di Castelnuovo A., de Gaetano G., Donati M.B., Iacoviello L. (2021). Different Anticoagulant Regimens, Mortality, and Bleeding in Hospitalized Patients with COVID-19: A Systematic Review and an Updated Meta-Analysis. Semin. Thromb. Hemost..

[24] Sadeghipour P., Talasaz A.H., Rashidi F., Sharif-Kashani B., Beigmohammadi M.T., Farrokhpour M., Sezavar S.H., Payandemehr P., Dabbagh A., Moghadam K.G. (2021). Effect of Intermediate-Dose vs Standard-Dose Prophylactic Anticoagulation on Thrombotic Events, Extracorporeal Membrane Oxygenation Treatment, or Mortality among Patients with COVID-19 Admitted to the Intensive Care Unit: The INSPIRATION Randomized Clinical Trial. JAMA.

[25] Perepu U.S., Chambers I., Wahab A., Ten Eyck P., Wu C., Dayal S., Sutamtewagul G., Bailey S.R., Rosenstein L.J., Lentz S.R. (2021). Standard prophylactic versus intermediate dose enoxaparin in adults with severe COVID-19: A multi-center, open-label, randomized controlled trial. J. Thromb. Haemost..

[26] Kumar D., Kaimaparambil V., Chandralekha S., Lalchandani J. (2022). Oral Rivaroxaban in the Prophylaxis of COVID-19 Induced Coagulopathy. J. Assoc. Physicians India.

[27] Connors J.M., Brooks M.M., Sciurba F.C., Krishnan J.A., Bledsoe J.R., Kindzelski A., Baucom A.L., Kirwan B.A., Eng H., Martin D. (2021). Effect of Antithrombotic Therapy on Clinical Outcomes in Outpatients with Clinically Stable Symptomatic COVID-19: The ACTIV-4B Randomized Clinical Trial. JAMA.

[28] Ramacciotti E., Barile Agati L., Calderaro D., Aguiar V.C.R., Spyropoulos A.C., de Oliveira C.C.C., Lins Dos Santos J., Volpiani G.G., Sobreira M.L., Joviliano E.E. (2022). Rivaroxaban versus no anticoagulation for post-discharge thromboprophylaxis after hospitalisation for COVID-19 (MICHELLE): An open-label, multicentre, randomised, controlled trial. Lancet.

[29] Cools F., Virdone S., Sawhney J., Lopes R.D., Jacobson B., Arcelus J.I., Hobbs F.D.R., Gibbs H., Himmelreich J.C.L., MacCallum P. (2022). Thromboprophylactic low-molecular-weight heparin versus standard of care in unvaccinated, at-risk outpatients with COVID-19 (ETHIC): An open-label, multicentre, randomised, controlled, phase 3b trial. Lancet Haematol..

[30] Wang T.Y., Wahed A.S., Morris A., Kreuziger L.B., Quigley J.G., Lamas G.A., Weissman A.J., Lopez-Sendon J., Knudson M.M., Siegal D.M. (2023). Effect of Thromboprophylaxis on Clinical Outcomes after COVID-19 Hospitalization. Ann. Intern. Med..

[31] Spyropoulos A.C., Goldin M., Giannis D., Diab W., Wang J., Khanijo S., Mignatti A., Gianos E., Cohen M., Sharifova G. (2021). Efficacy and Safety of Therapeutic-Dose Heparin vs. Standard Prophylactic or Intermediate-Dose Heparins for Thromboprophylaxis in High-risk Hospitalized Patients with COVID-19: The HEP-COVID Randomized Clinical Trial. JAMA Intern. Med..

[32] Smith K., Krajewski K.C., Krajewski M.P. (2020). Practical considerations in prevention and treatment of venous thromboembolism in hospitalized patients with COVID-19. Am. J. Health Syst. Pharm..

[33] Gunasekaran K., Rajasurya V., Devasahayam J., Singh Rahi M., Chandran A., Elango K., Talari G. (2020). A Review of the Incidence Diagnosis and Treatment of Spontaneous Hemorrhage in Patients Treated with Direct Oral Anticoagulants. J. Clin. Med..

[34] Rahi M.S., Pednekar P., Parmar G., Keibel L., Gunasekaran K., Amoah K., Winterbottom C. (2021). Spontaneous intercostal artery bleeding in a patient with alcohol-induced liver cirrhosis. Clin. Case Rep..

[35] Cuker A., Tseng E.K., Nieuwlaat R., Angchaisuksiri P., Blair C., Dane K., DeSancho M.T., Diuguid D.L., Griffin D.O., Kahn S.R. (2022). American Society of Hematology living guidelines on the use of anticoagulation for thromboprophylaxis in patients with COVID-19: January 2022 update on the use of therapeutic-intensity anticoagulation in acutely ill patients. Blood Adv..

[36] Schulman S., Sholzberg M., Spyropoulos A.C., Zarychanski R., Resnick H.E., Bradbury C.A., Broxmeyer L., Connors J.M., Falanga A., Iba T. (2022). ISTH guidelines for antithrombotic treatment in COVID-19. J. Thromb. Haemost..

[37] Barnes G.D., Burnett A., Allen A., Ansell J., Blumenstein M., Clark N.P., Crowther M., Dager W.E., Deitelzweig S.B., Ellsworth S. (2022). Thromboembolic prevention and anticoagulant therapy during the COVID-19 pandemic: Updated clinical guidance from the anticoagulation forum. J. Thromb. Thrombolysis.

[38] COVID-19 Treatment Guidelines Panel Coronavirus Disease 2019 (COVID-19) Treatment Guidelines. National Institue of Health. https://www.covid19treatmentguideliines.nih.gov/.

[39] Moores L.K., Tritschler T., Brosnahan S., Carrier M., Collen J.F., Doerschug K., Holley A.B., Iaccarino J., Jimenez D., LeGal G. (2022). Thromboprophylaxis in Patients With COVID-19: A Brief Update to the CHEST Guideline and Expert Panel Report. Chest.

